# Classical and Late‐Onset SOS/VOD After Allogeneic HSCT: A Japanese Transplant Registry Analysis

**DOI:** 10.1002/ajh.27715

**Published:** 2025-05-19

**Authors:** Kyoko Masuda, Keisuke Kataoka, Masatoshi Sakurai, Yuho Najima, Naonori Harada, Shoko Ukita, Naoyuki Uchida, Noriko Doki, Takahiro Fukuda, Masatsugu Tanaka, Hiroyuki Ohigashi, Jun Ishikawa, Satoshi Yoshihara, Masashi Sawa, Shuichi Ota, Yoshinobu Kanda, Tetsuya Nishida, Makoto Onizuka, Yoshiko Atsuta, Hideki Nakasone, Kimikazu Yakushijin

**Affiliations:** ^1^ Division of Hematology, Department of Medicine Keio University School of Medicine Tokyo Japan; ^2^ Division of Molecular Oncology National Cancer Center Research Institute Tokyo Japan; ^3^ Hematology Division, Tokyo Metropolitan Cancer and Infectious Diseases Center Komagome Hospital Tokyo Japan; ^4^ Department of Hematology Fuchu Hospital Izumi Japan; ^5^ Hematology, Graduate School of Medicine Osaka Metropolitan University Osaka Japan; ^6^ Biostatistics Unit, Clinical and Translational Research Center Keio University Hospital Tokyo Japan; ^7^ Department of Hematology Federation of National Public Service Personnel Mutual Aid Associations Toranomon Hospital Tokyo Japan; ^8^ Department of Hematopoietic Stem Cell Transplantation National Cancer Center Hospital Tokyo Japan; ^9^ Department of Hematology Kanagawa Cancer Center Yokohama Japan; ^10^ Department of Hematology Hokkaido University Hospital Sapporo Japan; ^11^ Department of Hematology Osaka International Cancer Institute Osaka Japan; ^12^ Department of Hematology Hyogo Medical University Hospital Hyogo Japan; ^13^ Department of Hematology and Oncology Anjo Kosei Hospital Anjo Japan; ^14^ Department of Hematology Sapporo Hokuyu Hospital Sapporo Japan; ^15^ Division of Hematology Jichi Medical University Shimotsuke Japan; ^16^ Department of Hematology Japanese Red Cross Aichi Medical Center Nagoya Daiichi Hospital Nagoya Japan; ^17^ Department of Hematology/Oncology Tokai University School of Medicine Isehara Japan; ^18^ Japanese Data Center for Hematopoietic Cell Transplantation Nagoya Japan; ^19^ Department of Registry Science for Transplant and Cellular Therapy Aichi Medical University School of Medicine Nagakute Japan; ^20^ Division of Hematology Jichi Medical University Saitama Medical Center Saitama Japan; ^21^ Division of Medical Oncology/Hematology, Department of Medicine Kobe University Graduate School of Medicine Kobe Japan

**Keywords:** allogeneic hematopoietic stem cell transplantation, classical SOS/VOD, late‐onset SOS/VOD, sinusoidal obstruction syndrome/veno‐occlusive disease

## Abstract

Sinusoidal obstruction syndrome/veno‐occlusive disease (SOS/VOD) is a lethal complication of allogeneic hematopoietic stem cell transplantation (allo‐HSCT). According to the 2016 European Society for Blood and Marrow Transplantation criteria, SOS/VOD is classified into classical SOS/VOD and late‐onset SOS/VOD, but their similarities and differences remain unclear. Here we retrospectively investigated the incidence, risk factors, and impact on transplant outcomes of classical and late‐onset SOS/VOD in 16 518 allo‐HSCT recipients using the Japanese nationwide registry data. The cumulative incidences of classical and late‐onset SOS/VOD were 2.5% and 2.2%, with a median onset of 13 and 42 days after transplantation, respectively. Both patients with classical (hazard ratio [HR], 3.45; 95% CI, 3.07–3.87) and late‐onset (HR, 3.98; 95% CI, 3.51–4.51) SOS/VOD had a significantly worse overall survival compared with those without. The risk factors for classical and late‐onset SOS/VOD are different. Hepatic comorbidities, high‐risk diseases, use of melphalan (MEL), and myeloablative conditioning are associated with both types of SOS/VOD. Whereas poor performance status, a prior history of transplantation, and positive hepatitis C virus are associated with only classical SOS/VOD, allo‐HSCT from cord blood or related human leukocyte antigen‐haploidentical donors, use of total body irradiation and busulfan (BU), and tacrolimus‐based graft‐versus‐host disease prophylaxis are associated with only late‐onset SOS/VOD. In particular, the incidence of late‐onset SOS/VOD is much higher in patients receiving both BU‐ and MEL‐containing conditioning regimens. These findings suggest that different monitoring and treatment approaches are necessary for allo‐HSCT recipients at high risk for classical and late‐onset SOS/VOD.

## 1 Introduction

Sinusoidal obstruction syndrome (SOS), also known as veno‐occlusive disease (VOD), is a potentially lethal complication of allogeneic hematopoietic stem cell transplantation (allo‐HSCT) [1, 2, 3, 4, 5]. The clinical characteristics of SOS/VOD consist of weight gain, fluid retention with ascites, painful hepatomegaly, and jaundice. Although SOS/VOD progressively resolves within a few weeks in most patients, the most severe forms result in multi‐organ failure (MOF), with a high mortality rate (> 80%). The incidence of SOS/VOD was previously reported to be 10%–15% but has declined to < 5% in recent years, probably due to safer transplant procedures and better prophylactic strategies [2, 3, 4, 5, 6, 7, 8, 9]. SOS/VOD is categorized into classical and late‐onset ones based on the onset time according to the European Society for Blood and Marrow Transplantation (EBMT) classification [3, 4, 5]. Classical SOS/VOD typically occurs within 21 days after allo‐HSCT and its diagnosis had been based on clinical and laboratory manifestations using the Baltimore or modified Seattle criteria before the revision of the EBMT criteria [10, 11]. The risk factors of classical SOS/VOD include transplant‐related factors (such as human leukocyte antigen [HLA]‐mismatched and unrelated donor, myeloablative conditioning [MAC], use of busulfan [BU], high‐dose total body irradiation [TBI], and second HSCT), patient and disease‐related factors (older age, lower performance status [PS], and advanced diseases), and hepatic‐related factors (pre‐existing liver disease including active viral hepatitis) [3, 4, 5].

On the other hand, late‐onset SOS/VOD occurs beyond 21 days after allo‐HSCT and has been increasingly described, accounting for at least one‐fourth of SOS [8, 9, 12]. Several single‐center studies have reported that late‐onset SOS/VOD may frequently occur in patients receiving BU‐containing regimens, [13, 14] and is associated with a worse prognosis similar to classical SOS/VOD [9]. However, as the sample size was limited in these studies, the clinical characteristics of late‐onset SOS/VOD and their differences from classical SOS/VOD have not been fully investigated.

Identification of risk factors is of particular interest, as the reduction of modifiable risk factors as well as close monitoring with possible early intervention with defibrotide is effective against patients at high risk for SOS/VOD [15, 16, 17, 18, 19]. Therefore, we retrospectively investigated the incidence, risk factors, and impact on transplant outcomes of classical and late‐onset SOS/VOD after allo‐HSCT using the Japanese nationwide registry data and evaluated the similarities and differences between classical and late‐onset SOS/VOD.

## 2 Methods

### 2.1 Study Design and Data Source

This is a retrospective study using a registry database, the Transplant Registry Unified Management Program (TRUMP), which is provided by the Japanese Society for Transplantation and Cellular Therapy (JSTCT) and Japanese Data Center for Hematopoietic Cell Transplantation (JDCHCT) [20, 21, 22]. Written informed consent was obtained from each patient at their respective institution. This study was approved by the data management committee of TRUMP and the ethics committee of Keio University School of Medicine (Tokyo, Japan) (Approval Number: 20226091).

### 2.2 Patients and Definitions

Based on the TRUMP database, this study included 16 518 patients aged 16 years or older who underwent allogeneic allo‐HSCT for hematologic disorders between 2017 and 2021. Patients with missing data were excluded. The diagnosis of SOS/VOD was established based on the EBMT criteria published in 2016 [3]. The criteria for classical SOS/VOD include the presence of bilirubin ≥ 2 mg/dL and two or more of the following criteria in the first 21 days after HSCT: painful hepatomegaly, weight gain > 5%, or ascites. The criteria for late‐onset SOS/VOD include (i) classical SOS/VOD, (ii) histologically proven SOS/VOD, or (iii) the presence of hemodynamical and/or ultrasound evidence of SOS/VOD and two or more of the following criteria beyond day 21: bilirubin ≥ 2 mg/dL, painful hepatomegaly, weight gain > 5%, or ascites. The TRUMP database includes information on classification (classical vs. late‐onset) and onset (time from transplantation) of SOS/VOD. When the SOS/VOD classification was not recorded, patients with SOS/VOD onset ≤ 21 days and > 21 days were classified into classical and late‐onset SOS/VOD, and those with the onset date of 22 days or later were defined as late‐onset SOS/VOD, respectively.

Regarding conditioning regimens, MAC and reduced‐intensity conditioning (RIC) were defined based on the criteria of the Center for International Blood and Marrow Transplant Research (CIBMTR) [23, 24]. Standard‐risk diseases were defined as acute myeloid leukemia (AML) in first or second complete remission, acute lymphoblastic leukemia (ALL) in first complete remission, chronic myeloid leukemia (CML) in chronic and accelerated phase, low‐risk myelodysplastic syndrome (MDS), myeloproliferative neoplasms (MPN), lymphomas with complete and partial response, and plasma cell neoplasms with complete, very good partial, and partial response. AML, ALL, CML, MDS, CML, lymphomas, plasma cell neoplasms in all other conditions were classified as high‐risk diseases. Other hematologic disorders, mainly consisting of bone marrow failure syndromes, were grouped into other hematologic disorders. As for comorbidities, patients were classified into five groups based on the hematopoietic cell transplantation comorbidity index (HCT‐CI) and the presence of hepatic comorbidity: low‐risk (HCT‐CI score of 0); intermediate‐risk (1, 2) without hepatic comorbidity; intermediate‐risk with hepatic comorbidity; high‐risk (≥ 3) without hepatic comorbidity; and high‐risk with hepatic comorbidity.

### 2.3 Statistical Analysis

The probability of overall survival (OS) was estimated using the Kaplan–Meier method and compared using the log‐rank test. Univariable analysis of OS was conducted using the Cox proportional hazards regression models, treating classical and late‐onset SOS/VOD as a time‐dependent variables. The probabilities of classical and late‐onset SOS/VOD were estimated using the cumulative incidence function, considering death as a competing risk [25]. Univariable and multivariable analyses of classical and late‐onset SOS/VOD incidences were conducted using Fine and Gray's proportional‐hazards model for subdistribution of a competing risk. Cumulative incidence curves were compared using Gray's test [26]. Risk factors for classical and late‐onset SOS/VOD were also evaluated using logistic regression analysis. All of the 16 518 patients were analyzed for classical SOS/VOD, whereas 15 625 patients who survived at 22 days after allo‐HSCT and did not develop classical SOS/VOD were analyzed for late‐onset SOS/VOD. The variables considered in univariable and multivariable analyses were age, Eastern Cooperative Oncology Group (ECOG) performance status (PS), HCT‐CI, disease risk, prior history of transplantation, donor source, conditioning regimen, use of TBI, BU, and melphalan (MEL) (in any dose), graft‐versus‐host disease (GVHD) prophylaxis, as well as HBV and HCV positivity. HBV and HCV positivity was defined by the presence of HBs antigen and HCV antibody, respectively. The effects of the combination of BU, MEL, and/or TBI use as well as their doses (when used outside their combinations) were evaluated in separate univariable analyses. In addition, previous use of gemtuzumab ozogamicin was also assessed in patients transplanted from 2020 onward, when its data collection started in the TRUMP database. All *p* values were two‐sided and considered statistically significant at < 0.05. All statistical analyses were performed with R 4.3.2 software.

## 3 Results

### 3.1 Patient Characteristics

A total of 16 518 patients were included in the analysis. Detailed patient background, disease status, and transplantation procedures are presented in Table 1. The median age of patients at allo‐HSCT was 53 years (range, 16–80) and 57% of patients were ≥ 50 years old. 9.8% of patients had poor PS, while 23% and 5.1% of patients had intermediate‐risk HCT‐CI (score 1–2) without and with hepatic comorbidity, respectively, and 13% and 4.8% had high‐risk HCT‐CI (score ≥ 3) without and with hepatic comorbidity, respectively. 53% and 43% of patients had standard‐risk and high‐risk diseases, while 2.1% and 2.1% had other hematologic diseases and bone marrow failure syndromes. 14% and 7.5% of patients underwent related HLA‐matched and HLA‐mismatched bone marrow (BM) or peripheral blood stem cell (PBSC) transplantation, which were mostly comprised of transplantation from partially mismatched donors, and 18% and 14% underwent unrelated HLA‐matched and partially HLA‐mismatched BM or PBSC transplantation, respectively, while 37% of patients received cord blood transplantation (CBT). 9.7% of patients received related HLA‐haploidentical PBSC transplantation, all of which used post‐transplant cyclophosphamide (PTCY). In terms of conditioning intensity, 45% and 55% of patients received RIC and MAC. TBI‐, BU‐, and MEL‐containing regimens were used in 63%, 45%, and 46% of patients, respectively. Regarding GVHD prophylaxis, 81% of patients received tacrolimus (TAC)‐based regimen, whereas 17% received cyclosporine (CSA)‐based regimen. Hepatitis B and C viruses were positive in 0.9% and 1.2% of patients. Gemtuzumab ozogamicin was used before transplantation in 1.3% of 6678 patients transplanted after 2020.

**TABLE 1:** Patient and transplant characteristics of 16 518 patients undergoing allo‐HSCT according to the presence of classical and late‐onset SOS/VOD.

	Overall (*n* = 16 518)	Classical SOS/VOD (*n* = 407)	Late‐onset SOS/VOD (*n* = 341)
Sex			
Female	6791 (41%)	145 (36%)	116 (34%)
Male	9726 (59%)	262 (64%)	225 (66%)
Unknown	1 (0.0%)	0 (0.0%)	0 (0.0%)
Age (years)			
< 50	7070 (43%)	184 (45%)	122 (36%)
≥ 50	9448 (57%)	223 (55%)	219 (64%)
ECOG PS			
0–1	14 873 (90%)	311 (76%)	292 (86%)
≥ 2	1624 (9.8%)	96 (24%)	49 (14%)
Unknown	21 (0.1%)	0 (0.0%)	0 (0.0%)
HCT‐CI			
0	9074 (55%)	162 (40%)	147 (43%)
1–2 (without hepatic comorbidity)	3562 (23%)	90 (22%)	71 (21%)
1–2 (with hepatic comorbidity)	839 (5.1%)	42 (10%)	28 (8.2%)
≥ 3 (without hepatic comorbidity)	2149 (13%)	55 (14%)	55 (16%)
≥ 3 (with hepatic comorbidity)	795 (4.8%)	47 (12%)	37 (11%)
Unknown	99 (0.6%)	11 (2.7%)	3 (0.9%)
Diagnosis			
AML	7050 (43%)	165 (41%)	157 (46%)
MDS	2357 (14%)	41 (10%)	45 (13%)
CML	371 (2.2%)	7 (1.7%)	4 (1.2%)
MPN	481 (2.9%)	23 (5.7%)	10 (2.9%)
ALL	2720 (16%)	83 (20%)	65 (19%)
Lymphoma	2662 (16%)	69 (17%)	56 (16%)
Plasma cell neoplasm	181 (1.1%)	3 (0.7%)	0 (0.0%)
Bone marrow failure syndromes	348 (2.1%)	6 (1.5%)	0 (0.0%)
Others	348 (2.1%)	10 (2.5%)	4 (1.2%)
Disease risk			
Standard	8753 (53%)	143 (35%)	136 (40%)
High	7069 (43%)	248 (61%)	201 (59%)
Other	696 (4.2%)	16 (3.9%)	4 (1.2%)
Number of transplants			
1	13 392 (81%)	275 (68%)	266 (78%)
≥ 2	3126 (19%)	132 (32%)	75 (22%)
Donor			
Matched related BM & PB	2356 (14%)	43 (11%)	15 (4.4%)
Mismatched related BM & PB	1242 (7.5%)	39 (9.6%)	22 (6.5%)
Matched unrelated BM & PB	3027 (18%)	56 (14%)	34 (10%)
Mismatched unrelated BM & PB	2248 (14%)	44 (11%)	26 (7.6%)
CB	6031 (37%)	178 (44%)	206 (60%)
Related haplo‐PTCY	1608 (9.7%)	47 (12%)	38 (11%)
Unknown	6 (0.0%)	0 (0.0%)	0 (0.0%)
Conditioning intensity			
RIC	7453 (45%)	197 (48%)	124 (36%)
MAC	9065 (55%)	210 (52%)	217 (64%)
TBI use	10 361 (63%)	264 (65%)	189 (55%)
BU use	7501 (45%)	163 (40%)	216 (63%)
MEL use	7649 (46%)	222 (55%)	222 (65%)
GVHD prophylaxis			
CSA‐based	2823 (17%)	68 (17%)	23 (7.0%)
TAC‐based	13 337 (81%)	327 (80%)	313 (92%)
Other	358 (2.2%)	12 (2.9%)	5 (1.5%)
HBV			
Negative	16 355 (99%)	401 (99%)	336 (99%)
Positive	163 (0.9%)	6 (1.5%)	5 (1.5%)
HCV			
Negative	16 313 (99%)	396 (97%)	338 (99%)
Positive	205 (1.2%)	11 (2.7%)	3 (0.9%)

Abbreviations: ALL, acute lymphoblastic leukemia; AML, acute myeloid leukemia; BM, bone marrow; BU, busulfan; CB, cord blood; CML, chronic myeloid leukemia; CSA, cyclosporine; CY, cyclophosphamide; ECOG, Eastern Cooperative Oncology Group; GVHD, graft versus host disease; HBV, hepatitis B virus; HCT‐CI, hematopoietic cell transplantation comorbidity index; HCV, hepatitis C virus; MAC, myeloablative conditioning; MDS, myelodysplastic syndrome; MEL, melphalan; MPN, myeloproliferative neoplasms; PB, peripheral blood; PS, performance status; PTCY, posttransplant cyclophosphamide; RIC, reduced‐intensity conditioning; TAC, tacrolimus; TBI, total body irradiation.

### 3.2 Cumulative Incidence of SOS/VOD

**FIGURE 1:**
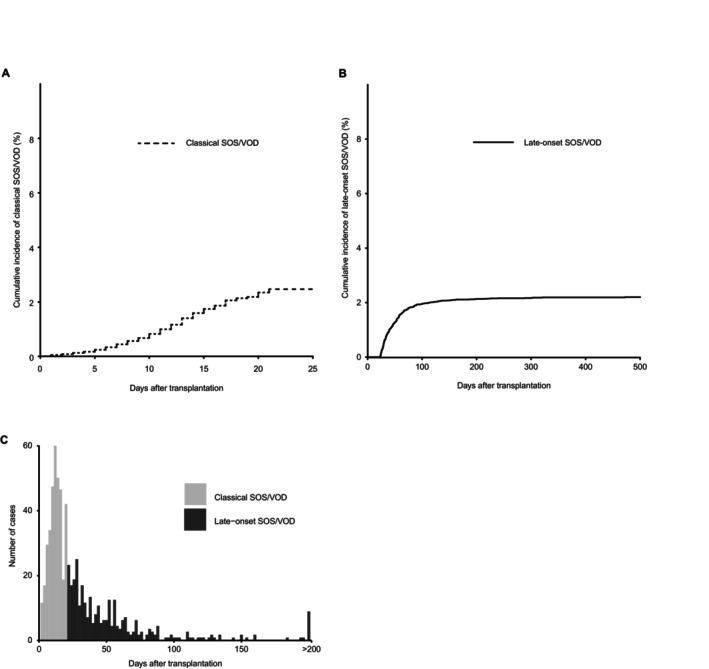
The incidences of classical and late‐onset SOS/VOD. The cumulative incidences of classical SOS/VOD (onset ≤ 21 days) in the entire cohort (*n* = 16 518) (A) and late‐onset SOS/VOD (onset > 21 days) in patients who survived at 22 days after allo‐HSCT and did not develop classical SOS/VOD (*n* = 15 625) (B). The time from allo‐HSCT to SOS/VOD onset for classical and late‐onset SOS/VOD (C).

In the entire cohort, the cumulative incidence of classical SOS/VOD (onset ≤ 21 days) was 2.5% (95% confidence interval [CI], 2.2%–2.7%) (Figure 1A). In 15 625 patients who survived at 22 days after allo‐HSCT and did not develop classical SOS/VOD, the cumulative incidence of late‐onset SOS/VOD was 2.2% (95% CI, 2.0%–2.4%) (Figure 1B). In total, the cumulative incidence of SOS/VOD was 4.6% (95% CI, 4.2%–4.9%) and late‐onset cases accounted for 46% of all SOS/VOD cases. The median duration from transplantation to SOS/VOD onset was 13 (interquartile range [IQR], 9–16) and 42 (IQR, 29–63) days for classical and late‐onset SOS/VOD, respectively (Figure 1C).

### 3.3 Impact of Classical and Late‐Onset SOS/VOD on OS

**FIGURE 2:**
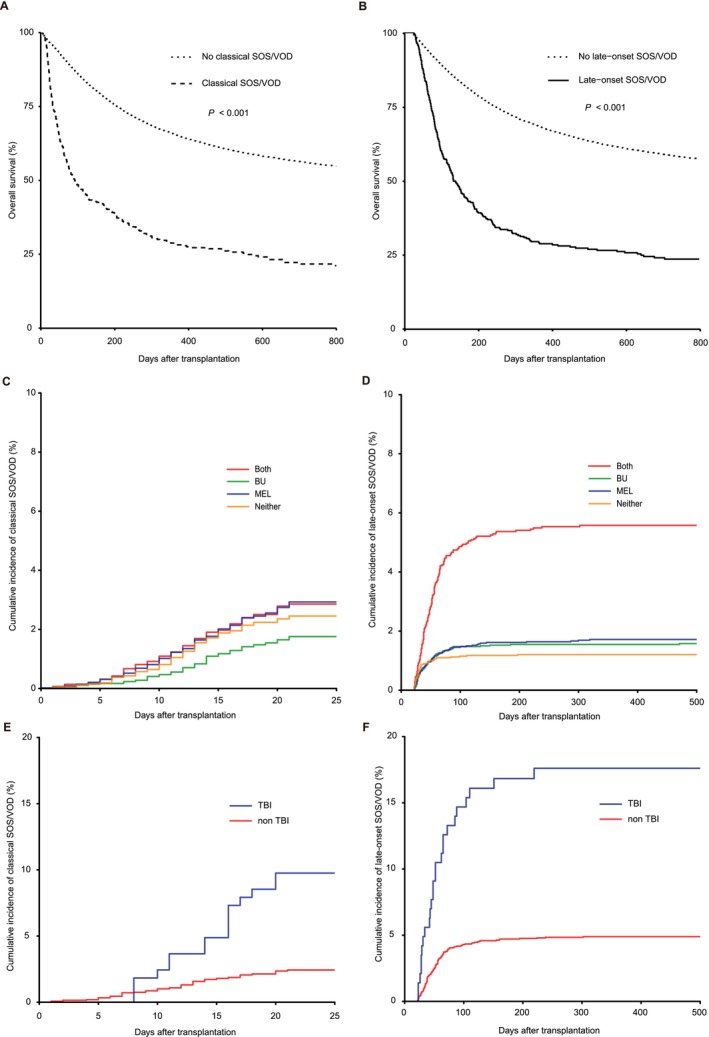
The impact of classical and late‐onset SOS/VOD on OS and the incidences of classical and late‐onset SOS/VOD according to BU, MEL, and TBI use. (A, B) The probability of OS in patients who developed (*n* = 407) and did not develop (*n* = 16 111) classical SOS/VOD in the entire cohort (*n* = 16 518) (A) and in patients who developed (*n* = 341) and did not develop (*n* = 15 284) late‐onset SOS/VOD in patients who survived at 22 days after allo‐HSCT and did not develop classical SOS/VOD (*n* = 15 625) (B). (C) The cumulative incidences of classical SOS/VOD in patients receiving BU‐ (*n* = 4667), MEL‐ (*n* = 4815), both‐ (*n* = 2834), or neither (*n* = 4202)‐containing conditioning regimens in the entire cohort (*n* = 16 518). (D) The cumulative incidences of late‐onset SOS/VOD in patients receiving BU‐ (*n* = 4490), MEL‐ (*n* = 4488), both‐ (*n* = 2643), or neither (*n* = 4004)‐containing conditioning regimens in patients who survived at 22 days after allo‐HSCT and did not develop classical SOS/VOD (*n* = 15 625). (E) The cumulative incidences of classical SOS/VOD in patients who underwent (*n* = 164) and did not undergo TBI (*n* = 2670) in patients who received both BU and MEL (*n* = 2834). (F) The cumulative incidences of classical SOS/VOD in patients who underwent (*n* = 143) and did not undergo TBI (*n* = 2500) in patients who received both BU and MEL, survived beyond 22 days after allo‐HSCT, and did not develop classical SOS/VOD (*n* = 2643). [Color figure can be viewed at wileyonlinelibrary.com]

At the time of the last follow‐up, 9317 of 16 518 patients were alive. The median follow‐up periods for survivors were 779 (range, 4–2065) days after allo‐HSCT. In the entire cohort, patients who developed classical SOS/VOD had a worse OS (48.5%; 95% CI, 43.8%–53.6% at 100 days and 38.6%; 95% CI, 34.1%–43.7% at 200 days) than those without classical SOS/VOD (86.1%; 95% CI, 85.6%–86.6% at 100 days and 75.5%; 95% CI, 74.9%–76.2% at 200 days) (*p* < 0.001) (Figure 2A). In the univariable Cox proportional hazards regression model treating classical SOS/VOD as a time‐dependent variable, patients with classical SOS/VOD had a significantly worse OS compared with those without (hazard ratio [HR], 3.45; 95% CI, 3.07–3.87; *p* < 0.001). Similarly, in 15 625 patients who survived at 22 days after allo‐HSCT and did not develop classical SOS/VOD, patients who developed late‐onset SOS/VOD had an inferior OS (60.1%; 95% CI, 55.1%–65.5% at 100 days and 39.3%; 95% CI, 34.3%–44.9% at 200 days) than those without late‐onset SOS/VOD (89.4%; 95% CI, 88.9%–89.9% at 100 days and 78.7%; 95% CI, 78.0%–79.4% at 200 days) (*p* < 0.001) (Figure 2B). In the Cox proportional hazards regression model, patients with late‐onset SOS/VOD had a significantly worse OS compared with those without (HR, 3.98; 95% CI, 3.51–4.51; *p* < 0.001). These observations suggest that SOS/VOD is associated with worse survival irrespective of its onset time.

Treatment contents were almost equivalent between classical and late‐onset SOS/VOD, including defibrotide, although fresh frozen plasma was more frequently used in patients with late‐onset SOS/VOD (Table S1). The causes of death in 303 and 252 patients who developed classical and late‐onset SOS/VOD, respectively, are presented in Table S2. The causes of death were almost comparable between patients who developed classical and late‐onset SOS/VOD.

### 3.4 Risk Factors for Classical and Late‐Onset SOS/VOD

In univariable competing risk analyses, poor PS, intermediate‐risk and high‐risk HCT‐CI (regardless of hepatic comorbidity), high‐risk diseases, a prior history of transplantation, HSCT from CB or related partially HLA‐mismatched and HLA‐haploidentical donors, use of MEL, and positive HCV were significantly associated with a higher incidence of classical SOS/VOD (Table 2). On the other hand, the use of BU was significantly associated with a lower incidence of classical SOS/VOD.

**TABLE 2:** Univariable and multivariable analyses for risk factors of classical and late‐onset SOS/VOD: Competing‐risk regression model.

	Classical SOS/VOD						Late‐onset SOS/VOD					
	Univariable			Multivariable			Univariable			Multivariable		
	HR	95% CI	*p*	HR	95% CI	*p*	HR	95% CI	*p*	HR	95% CI	*p*
Age (years)												
< 50	1			1			1			1		
≥ 50	0.91	0.74–1.10	0.320	0.95	0.76–1.18	0.630	1.36	1.09–1.70	0.007	1.03	0.82–1.30	0.790
ECOG PS												
0–1	1			1			1			1		
≥ 2	2.89	2.30–3.63	< 0.001	2.03	1.58–2.60	< 0.001	1.80	1.33–2.43	< 0.001	1.33	0.97–1.81	0.078
HCT‐CI												
0	1			1			1			1		
1–2 (without hepatic comorbidity)	1.42	1.10–1.84	0.007	1.29	1.00–1.67	0.049	1.26	0.95–1.68	0.110	1.11	0.83–1.47	0.490
1–2 (with hepatic comorbidity)	2.84	2.03–3.99	< 0.001	2.54	1.81–3.55	< 0.001	2.17	1.45–3.25	< 0.001	1.83	1.23–2.73	0.003
≥ 3 (without hepatic comorbidity)	1.44	1.06–1.96	0.019	1.27	0.92–1.73	0.140	1.64	1.20–2.23	0.002	1.32	0.97–1.81	0.080
≥ 3 (with hepatic comorbidity)	3.39	2.45–4.69	< 0.001	2.61	1.87–3.63	< 0.001	3.27	2.28–4.69	< 0.001	2.59	1.80–3.74	< 0.001
Disease risk												
Standard	1			1			1			1		
High	2.17	1.77–2.66	< 0.001	1.75	1.40–2.18	< 0.001	1.93	1.56–2.40	< 0.001	1.39	1.11–1.73	0.004
Other	1.41	0.84–2.37	0.190	0.99	0.55–1.78	0.970	0.38	0.14–1.04	0.630	0.54	0.20–1.48	0.230
Number of transplants												
1	1			1			1			1		
≥ 2	2.08	1.69–2.56	< 0.001	1.58	1.24–2.00	< 0.001	1.30	1.00–1.68	0.046	1.10	0.85–1.44	0.470
Donor												
Matched related BM & PB	1			1			1			1		
Mismatched related BM & PB	1.73	1.12–2.67	0.013	1.13	0.69–1.85	0.630	2.95	1.53–5.69	0.001	1.67	0.80–3.52	0.180
Matched unrelated BM & PB	1.01	0.68–1.51	0.950	1.19	0.77–1.83	0.440	1.78	0.97–3.27	0.063	1.15	0.59–2.24	0.670
Mismatched unrelated BM & PB	1.07	0.70–1.63	0.750	1.20	0.76–1.89	0.430	1.84	0.98–3.48	0.060	1.17	0.58–2.37	0.650
CB	1.62	1.16–2.27	0.004	1.31	0.91–1.91	0.150	5.70	3.37–9.63	< 0.001	2.74	1.51–4.98	< 0.001
Related haplo‐PTCY	1.61	1.06–2.43	0.024	1.61	1.00–2.60	0.050	3.85	2.11–7.00	< 0.001	2.42	1.23–4.75	0.010
Conditioning intensity												
RIC	1			1			1			1		
MAC	0.88	0.72–1.06	0.180	1.32	1.03–1.68	0.026	1.39	1.12–1.74	0.003	1.90	1.41–2.55	< 0.001
TBI use	1.10	0.89–1.34	0.380	1.18	0.90–1.55	0.230	0.73	0.59–0.90	0.004	2.11	1.46–3.04	< 0.001
BU use	0.80	0.66–0.98	0.028	0.81	0.61–1.07	0.140	2.07	1.66–2.58	< 0.001	2.42	1.73–3.40	< 0.001
MEL use	1.40	1.15–1.70	< 0.001	1.29	1.01–1.64	0.040	2.23	1.79–2.79	< 0.001	2.67	1.92–3.70	< 0.001
GVHD prophylaxis												
CSA‐based	1			1			1			1		
TAC‐based	1.02	0.78–1.32	0.900	0.79	0.59–1.06	0.110	2.94	1.92–4.48	< 0.001	1.93	1.21–3.08	0.006
Other	1.40	0.76–2.58	0.280	1.16	0.62–2.17	0.650	1.85	0.70–4.87	0.210	1.61	0.60–4.33	0.350
HBV												
Negative	1			1			1			1		
Positive	1.51	0.68–3.38	0.320	1.43	0.64–3.22	0.380	1.52	0.63–3.69	0.350	1.37	0.56–3.37	0.500
HCV												
Negative	1			1			1					
Positive	2.26	1.24–4.12	0.008	2.20	1.20–4.02	0.011	0.76	0.24–2.35	0.630	0.71	0.23–2.13	0.540

Abbreviations: BM, bone marrow; BU, busulfan; CB, cord blood; CI, confidence interval; CSA, cyclosporine; CY, cyclophosphamide; ECOG, Eastern Cooperative Oncology Group; GVHD, graft versus host disease; HBV, hepatitis B virus; HCT‐CI, hematopoietic cell transplantation comorbidity index; HCV, hepatitis C virus; HR, hazard ratio; MAC, myeloablative conditioning; MEL, melphalan; PB, peripheral blood; PS, performance status; PTCY, posttransplant cyclophosphamide; RIC, reduced‐intensity conditioning; TAC, tacrolimus; TBI, total body irradiation.

In multivariable competing risk analysis, poor PS, intermediate‐risk HCT‐CI (regardless of hepatic comorbidity), high‐risk HCT‐CI with hepatic comorbidity, high‐risk diseases, a prior history of transplantation, MAC, use of MEL, and positive HCV were independently associated with a higher incidence of classical SOS/VOD.

In univariable competing risk analyses, older age, poor PS, intermediate‐risk and high‐risk HCT‐CI (regardless of hepatic comorbidity), high‐risk diseases, a prior history of transplantation, HSCT from CB or related partially HLA‐mismatched and HLA‐haploidentical donors, MAC, use of BU and MEL, and TAC‐based GVHD prophylaxis were significantly associated with a higher incidence of late‐onset SOS/VOD. On the other hand, the use of TBI was significantly associated with a lower incidence of late‐onset SOS/VOD.

In multivariable competing risk analysis, intermediate‐risk HCT‐CI with hepatic comorbidity, high‐risk HCT‐CI with hepatic comorbidity, high‐risk diseases, HSCT from CB or related haploidentical donors, MAC, use of TBI, BU, and MEL, and TAC‐based GVHD prophylaxis were independently associated with a higher incidence of late‐onset SOS/VOD.

Similar results were obtained when logistic regression analysis was used (Table S3).

In a univariable analysis assessing the impact of gemtuzumab ozogamicin in patients transplanted after 2020, its previous use was significantly associated with a higher incidence of classical SOS/VOD (*p* < 0.001), whereas there was no association with late‐onset SOS/VOD (*p* = 0.538) (Figure S1).

### 3.5 Combined Effect of BU, MEL, and TBI Use on SOS/VOD Incidence

As the two alkylating agents, BU and MEL, were associated with a higher incidence of late‐onset SOS/VOD, we evaluated the combined effect of BU and MEL on the incidences of classical and late‐onset SOS/VOD. The cumulative incidences of classical SOS/VOD were almost comparable: 1.8% (95% CI, 1.4%–2.1%), 2.9% (95% CI, 2.5%–3.4%), 2.9% (95% CI, 2.2%–3.5%), and 2.5% (95% CI, 2.0%–2.9%) for patients receiving BU‐, MEL‐, both‐, or neither‐containing conditioning regimens, respectively (*p* = 0.001) (Figure 2C). On the other hand, the cumulative incidences of late‐onset SOS/VOD were 1.6% (95% CI, 1.2%–1.9%), 1.7% (95% CI, 1.3%–2.1%), 5.6% (95% CI, 4.7%–6.5%), and 1.2% (95% CI, 0.9%–1.6%) for patients receiving BU‐, MEL‐, both‐, or neither‐containing conditioning regimens, respectively (*p* < 0.001) (Figure 2D).

In addition, we evaluated the impact of TBI on the cumulative incidences of classical and late‐onset SOS/VOD in patients receiving both BU and MEL. The cumulative incidence of classical SOS/VOD tended to be higher in patients who received TBI (9.8%; 95% CI, 5.2%–14.3%) compared with those who did not receive TBI (2.4%; 95% CI, 1.9%–3.0%) (*p* < 0.001) (Figure 2E). The cumulative incidence of late‐onset SOS/VOD was much higher in those who received TBI (17.6%; 95% CI, 11.3%–23.9%) than in those who did not receive TBI (4.9%; 95% CI, 4.0%–5.7%) (*p* < 0.001) (Figure 2F).

On the other hand, there was no association between a higher dose of BU (> 6.4 mg/kg), MEL (> 140 mg/kg), or TBI (≥ 8 Gy) and incidences of classical and late‐onset SOS/VOD when used outside their combinations (Figure S2).

## 4 Discussion

By analyzing 16 518 adult patients using the Japanese registry database, we revealed that the cumulative incidences of classical and late‐onset SOS/VOD are 2.5% and 2.2%, respectively, consistent with those reported in recent studies [8, 9]. The proportion of late‐onset cases of all SOS/VOD cases was relatively high (46%), which is in line with recent reports, [9, 12] although lower proportions have been described [8]. In addition, we have provided reliable estimates for the transplant outcomes of patients who developed classical (OS of 38.6% at 200 days compared with 75.5% for patients without) and late‐onset SOS/VOD (OS of 39.3% at 200 days compared with 78.7% for patients without). A comparable adverse impact of classical and late‐onset SOS/VOD suggests that clinicians should consider close monitoring with possible early intervention even beyond 21 days after allo‐HSCT for patients at high risk for late‐onset SOS/VOD.

The most prominent finding in this study is the substantial differences of risk factors for classical and late‐onset SOS/VOD. Although intermediate‐risk and high‐risk HCT‐CI with hepatic comorbidity, high‐risk diseases, use of MEL, and MAC are associated with both types of SOS/VOD, poor PS, intermediate‐risk HCT‐CI without hepatic comorbidity, a prior history of transplantation, and positive HCV are associated with only classical SOS/VOD, whereas HSCT from CB or related HLA‐haploidentical donors, use of TBI and BU, and TAC‐based GVHD prophylaxis are associated with only late‐onset SOS/VOD. In particular, the incidence of late‐onset SOS/VOD is much higher in patients receiving both BU‐ and MEL‐containing conditioning regimens. In addition, the incidence is further higher in patients undergoing TBI among those who received both BU and MEL. Risk factors for classical SOS/VOD are consistent with those found in previous studies, [3, 4, 5] although use of BU and TBI (even at high dose) are not associated with its development in our analysis, maybe due to increased use of RIC and changes in conditioning regimens. Risk factors for late‐onset SOS/VOD include many modifiable ones which can be reduced for improving patient outcomes [4]. Although HLA‐haploidentical PBSC transplantation with PTCY was reported to show no increased incidence of SOS/VOD, [27, 28] related HLA‐haploidentical donor is associated with late‐onset SOS/VOD, probably due to the more common use of conditioning regimens incorporating alkylating agent and PTCY [29, 30, 31].

These observations provide several important implications. Clinically, given defibrotide is effective against moderate to severe SOS/VOD and should be initiated as soon as possible in those patients, [12, 16, 32] careful monitoring over a longer period is needed for patients at high risk for late‐onset SOS/VOD. For the prophylaxis of SOS/VOD, administration of ursodeoxycholic acid is recommended in adult patients [33, 34, 35]. Moreover, prophylactic use of defibrotide has been evaluated in several studies; however, the efficacy of its preventive application has yet to be established [15, 36, 37]. Therefore, the duration of prophylaxis should be adjusted according to the presence of risk factors for classical and late‐onset SOS/VOD.

As for the pathophysiology of SOS/VOD, conditioning regimens cause direct damage to sinusoidal endothelial cells and hepatocytes [38, 39]. Damaged endothelial cells release inflammatory cytokines and express adhesion molecules, leading to the activation and recruitment of leukocytes and platelets. The injured endothelial cells and surrounding hepatocytes undergo apoptosis, resulting in detachment and embolization within the sinusoids. This obstruction impedes blood flow, causing increased sinusoidal pressure and portal hypertension [2]. Different risk factors between classical and late‐onset SOS/VOD suggest that such pathophysiology and/or evolution process may be different between them. Although the current classification of classical and late‐onset SOS/VOD is based on the onset time (21 days after allo‐HSCT) [3, 4], it may be more clinically meaningful to categorize them according to not only the onset time but also underlying pathophysiology and risk factors.

The limitations of this study were its retrospective nature. In addition, the criteria for diagnosis for SOS/VOD were updated recently, particularly for refining the previous classification and distinguishing probable, clinical, and proven SOS/VOD at diagnosis [4]. Therefore, further investigation using the latest EBMT criteria would be needed. Moreover, the database did not include information on the severity grading of SOS/VOD, which is reported to be strongly associated with worse survival after allo‐HSCT [12, 40]. Although previous use of gemtuzumab ozogamicin is likely associated with an increased risk of classical SOS/VOD only, a large‐scale study is required to confirm the result. Furthermore, other information, such as the details of prophylaxis for SOS/VOD and previous use of inotuzumab ozogamicin, is not available.

In conclusion, our study demonstrated that although the current incidence of SOS/VOD is relatively low, late‐onset cases constitute more than one‐third of all SOS/VOD cases and are associated with worse survival like classical SOS/VOD. More importantly, we identified risk factors for late‐onset SOS/VOD and clarified the differences from those for classical SOS/VOD. Particularly, risk factors for late‐onset SOS/VOD include many modifiable ones, such as HSCT from CB or related HLA‐haploidentical donors, use of TBI and BU, and TAC‐based GVHD prophylaxis. These findings suggest that different monitoring and treatment strategies would be needed for patients at high risk for classical and late‐onset SOS/VOD.

## Author Contributions

K.M. and K.K. designed the study, analyzed the data, and wrote the manuscript. M.S. assisted in interpreting the results. S.U. assisted with the statistical analyses. Y.N., N.H., N.U., N.D., T.F., M.T., H.O., J.I., S.Y., M.S., S.O., Y.K., T.N., M.O., Y.A., H.N., and K.Y. collected data. All authors reviewed the final version of the manuscript.

## Conflicts of Interest

K.K. received honoraria from Ono Pharmaceutical, Eisai, Astellas Pharma, Novartis, Chugai Pharmaceutical, AstraZeneca, Sumitomo Pharma, Kyowa Kirin, Janssen Pharmaceutical, Takeda Pharmaceutical, Otsuka Pharmaceutical, SymBio Pharmaceuticals, Bristol Myers Squibb, Pfizer, Nippon Shinyaku, Daiichi Sankyo, Alexion Pharmaceuticals, AbbVie, Meiji Seika Pharma, Sanofi, Sysmex, Mundipharma, Incyte Corporation, and Kyorin Pharmaceutical. K.K. received research support from Otsuka Pharmaceutical, Chordia Therapeutics, Chugai Pharmaceutical, Takeda Pharmaceutical, and Meiji Seika Pharma. K.K. received scholarship from Asahi Kasei Pharma, Eisai, Otsuka Pharmaceutical, Ono Pharmaceutical, Kyowa Kirin, Shionogi, Takeda Pharmaceutical, Sumitomo Dainippon Pharma, Chugai Pharmaceutical, Teijin Pharma, Japan Blood Products Organization, Mochida Pharmaceutical, JCR Pharmaceuticals, and Nippon Shinyaku. K.K. owns stock in Asahi Genomics. K.K. has a patent for Genetic alterations as a biomarker in T‐cell lymphomas and a patent for PD‐L1 abnormalities as a predictive biomarker for immune checkpoint blockade therapy. The other authors declare no competing interests. MSakurai owns stock in Celaid Therapeutics. N.H. received honoraria from Nippon Shinyaku. MSawa received honoraria from Nippon Shinyaku. S.O. received honoraria from Novartis, AstraZeneca, Janssen Pharmaceutical, Takeda Pharmaceutical, PharmaEssensia, Bristol Myers Squibb, AbbVie, Sanofi, AstraZeneca, and Amgen. Y.A. received lecture fee from Otsuka Pharmaceutical, Chugai Pharmaceutical, Novartis Pharma, and AbbVie. Y.A. received consultant fee from JCR Pharmaceuticals and Kyowa Kirin. Y.A. received honoraria from Meiji Seika Pharma. K.Y. received honoraria from Nippon Shinyaku and JAZZ pharmaceuticals.

## Supporting information


**Figure S1.** The impact of previous use of gemtuzumab ozogamicin on the incidences of classical and late‐onset SOS/VOD. (A) The cumulative incidences of classical SOS/VOD in patients receiving gemtuzumab ozogamicin (*n* = 88) or not (*n* = 6590) before transplantation among patients who were transplanted after 2020 (*n* = 6678). (B) The cumulative incidences of late‐onset SOS/VOD in patients receiving gemtuzumab ozogamicin (*n* = 74) or not (*n* = 6201) before transplantation among patients who were transplanted after 2020 (*n* = 6275).

**Figure S2.** Cumulative incidences of classical and late‐onset SOS/VOD according to the dose of BU, MEL, and TBI used in conditioning regimens. (A) The cumulative incidences of classical SOS/VOD in patients receiving BU ≤ 6.4 mg/kg (*n* = 229) or BU > 6.4 mg/kg (*n* = 1692) among patients who received BU‐containing conditioning regimens without MEL or TBI use (*n* = 1922). Information on BU dose was not available for 1 patient. (B) The cumulative incidences of late‐onset SOS/VOD in patients receiving BU ≤ 6.4 mg/kg (*n* = 213) or BU > 6.4 mg/kg (*n* = 1641) among patients who received BU‐containing conditioning regimens without MEL or TBI use, survived at 22 days after allo‐HSCT, and did not develop classical SOS/VOD (*n* = 1854). (C) The cumulative incidences of classical SOS/VOD in patients receiving MEL ≤ 140 mg/kg (*n* = 1244) or MEL > 140 mg/kg (*n* = 113) among patients who received MEL‐containing conditioning regimens without BU or TBI use (*n* = 1357). (D) The cumulative incidences of late‐onset SOS/VOD in patients receiving MEL ≤ 140 mg/kg (*n* = 1127) or MEL > 140 mg/kg (*n* = 108) among patients who received MEL‐containing conditioning regimens without BU or TBI use, survived at 22 days after allo‐HSCT, and did not develop classical SOS/VOD (*n* = 1235). (E) The cumulative incidences of classical SOS/VOD in patients receiving TBI < 8 Gy (*n* = 599) or TBI ≥ 8 Gy (*n* = 3395) among patients who received TBI‐containing conditioning regimens without BU or MEL (*n* = 3994). (F) The cumulative incidences of late‐onset SOS/VOD in patients receiving TBI < 8 Gy (*n* = 551) or TBI ≥ 8 Gy (*n* = 3274) among patients who received TBI‐containing conditioning regimens without BU or MEL, survived at 22 days after allo‐HSCT, and did not develop classical SOS/VOD (*n* = 3825).

Tables S1‐S3.

## Data Availability

The data of this study are not publicly available due to ethical restrictions that they exceed the scope of the recipient/donor's consent for research use in the registry.
