# Supplementary figures and images for: Classical and Late‐Onset SOS/VOD After Allogeneic HSCT: A Japanese Transplant Registry Analysis
Source: Am J Hematol. 2025 May 19;100(8):1283–94. doi: 10.1002/ajh.27715 (PMC12232550; doi:10.1002/ajh.27715)

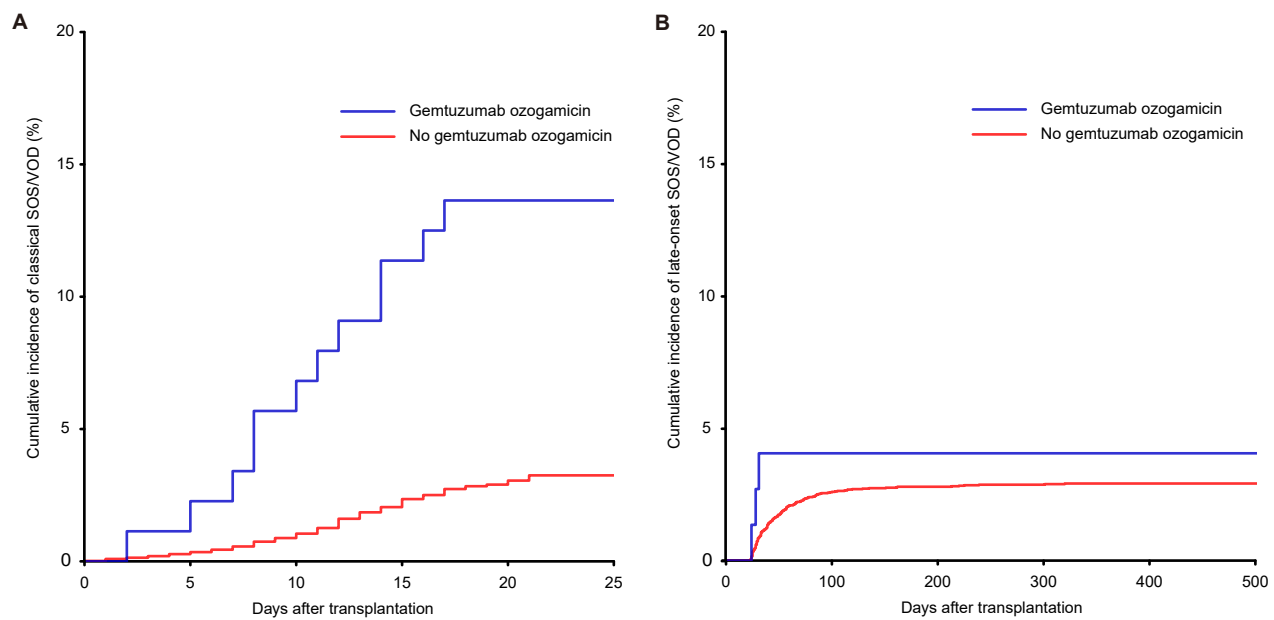

Supplement: Supplementary file 1 — Figure S1. The impact of previous use of gemtuzumab ozogamicin on the incidences of classical and late‐onset SOS/VOD. (A) The cumulative incidences of classical SOS/VOD in patients receiving gemtuzumab ozogamicin (n = 88) or not (n = 6590) before transplantation among patients who were transplanted after 2020 (n = 6678). (B) The cumulative incidences of late‐onset SOS/VOD in patients receiving gemtuzumab ozogamicin (n = 74) or not (n = 6201) before transplantation among patients who were transplanted after 2020 (n = 6275). [file AJH-100-1283-s001.pdf]

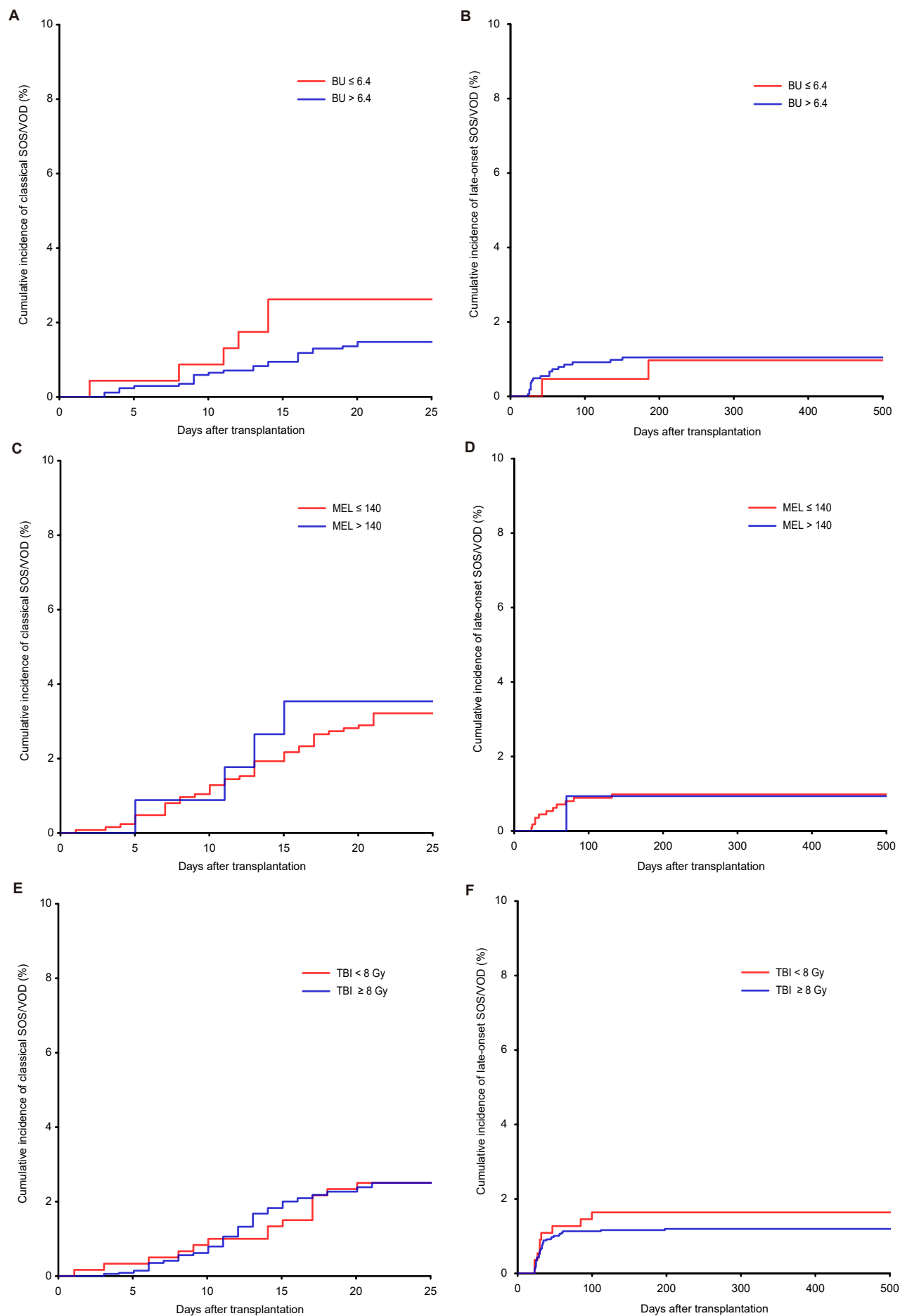

Supplementary Figure 2

Supplement: Supplementary file 2 — Figure S2. Cumulative incidences of classical and late‐onset SOS/VOD according to the dose of BU, MEL, and TBI used in conditioning regimens. (A) The cumulative incidences of classical SOS/VOD in patients receiving BU ≤ 6.4 mg/kg (n = 229) or BU > 6.4 mg/kg (n = 1692) among patients who received BU‐containing conditioning regimens without MEL or TBI use (n = 1922). Information on BU dose was not available for 1 patient. (B) The cumulative incidences of late‐onset SOS/VOD in patients receiving BU ≤ 6.4 mg/kg (n = 213) or BU > 6.4 mg/kg (n = 1641) among patients who received BU‐containing conditioning regimens without MEL or TBI use, survived at 22 days after allo‐HSCT, and did not develop classical SOS/VOD (n = 1854). (C) The cumulative incidences of classical SOS/VOD in patients receiving MEL ≤ 140 mg/kg (n = 1244) or MEL > 140 mg/kg (n = 113) among patients who received MEL‐containing conditioning regimens without BU or TBI use (n = 1357). (D) The cumulative incidences of late‐onset SOS/VOD in patients receiving MEL ≤ 140 mg/kg (n = 1127) or MEL > 140 mg/kg (n = 108) among patients who received MEL‐containing conditioning regimens without BU or TBI use, survived at 22 days after allo‐HSCT, and did not develop classical SOS/VOD (n = 1235). (E) The cumulative incidences of classical SOS/VOD in patients receiving TBI < 8 Gy (n = 599) or TBI ≥ 8 Gy (n = 3395) among patients who received TBI‐containing conditioning regimens without BU or MEL (n = 3994). (F) The cumulative incidences of late‐onset SOS/VOD in patients receiving TBI < 8 Gy (n = 551) or TBI ≥ 8 Gy (n = 3274) among patients who received TBI‐containing conditioning regimens without BU or MEL, survived at 22 days after allo‐HSCT, and did not develop classical SOS/VOD (n = 3825). [file AJH-100-1283-s003.pdf]
