# Supplementary material for: Classical and Late‐Onset SOS/VOD After Allogeneic HSCT: A Japanese Transplant Registry Analysis
Source: Am J Hematol. 2025 May 19;100(8):1283–94. doi: 10.1002/ajh.27715 (PMC12232550; doi:10.1002/ajh.27715)
Supplement: Supplementary file 3 — Tables S1‐S3. [file AJH-100-1283-s002.docx]

Supplementary Table 1. Treatment of classical and late-onset SOS/VOD.

| **Supplementary Table 1. Treatment of classical and late-onset SOS/VOD**. | | | |
| --- | --- | --- | --- |
|  | **All SOS**  **(n = 748)** | **Classical SOS/VOD**  **(n = 407)** | **Late-onset SOS/VOD**  **(n = 341)** |
| **Defibrotide** | 380 (51%) | 221 (54%) | 159 (47%) |
| **Recombinant human soluble thrombomodulin** | 341 (46%) | 172 (42%) | 169 (50%) |
| **Ursodeoxycholic acid** | 310 (41%) | 173 (43%) | 137 (40%) |
| **Fresh frozen plasma** | 259 (35%) | 118 (29%) | 141 (41%) |
| **Antithrombin III** | 109 (15%) | 55 (14%) | 54 (16%) |
| **Heparin** | 102 (14%) | 55 (14%) | 47 (14%) |

Supplementary Table 2. Causes of death in 303 and 252 patients who developed classical and late-onset SOS/VOD.

| **Supplementary Table 2. Causes of death in 303 and 252 patients who developed classical and late-onset SOS/VOD**. | | | |
| --- | --- | --- | --- |
|  | **Classical SOS/VOD**  **n = 303 (%)** | **Late-onset SOS/VOD**  **n = 252 (%)** |  |
| SOS/VOD | 72 (24%) | 64 (25%) |  |
| Relapse | 66 (22%) | 39 (15%) |  |
| Infection | 59 (19%) | 49 (19%) |  |
| Organ failure | 51 (17%) | 52 (21%) |  |
| GVHD | 19 (6%) | 22 (9%) |  |
| Other | 36 (12%) | 26 (10%) | *P* = 0.352 (Fisher's exact test) |

*GVHD*, graft-versus-host disease.

Supplementary Table 3. Univariable and multivariable analyses for risk factors of classical and late-onset SOS/VOD: logistic regression model.

| **Supplementary Table 3. Univariable and multivariable analyses for risk factors of classical and late-onset SOS/VOD: logistic regression model**. | | | | | | | | | | | |  |  |  |  |
| --- | --- | --- | --- | --- | --- | --- | --- | --- | --- | --- | --- | --- | --- | --- | --- |
|  | **Classical SOS/VOD** | | | | | | |  | **Late-onset SOS/VOD** | | | | | | |
|  | **Univariable** | | |  | **Multivariable** | | |  | **Univariable** | | |  | **Multivariable** | | |
|  | **OR** | **95% CI** | ***P* value** |  | **OR** | **95% CI** | ***P* value** |  | **OR** | **95% CI** | ***P* value** |  | **OR** | **95% CI** | ***P* value** |
| **Age (years)** |  |  |  |  |  |  |  |  |  |  |  |  |  |  |  |
|  | 1 |  |  |  | 1 |  |  |  | 1 |  |  |  | 1 |  |  |
| ≥ 50 | 0.90 | 0.74-1.10 | 0.321 |  | 0.95 | 0.76-1.19 | 0.650 |  | 1.36 | 1.09-1.70 | 0.006 |  | 1.03 | 0.80-1.33 | 0.807 |
| **ECOG PS** |  |  |  |  |  |  |  |  |  |  |  |  |  |  |  |
| 0-1 | 1 |  |  |  | 1 |  |  |  | 1 |  |  |  | 1 |  |  |
| ≥ 2 | 2.94 | 2.33-3.72 | <0.001 |  | 2.05 | 1.58-2.65 | <0.001 |  | 1.82 | 1.34-2.48 | <0.001 |  | 1.33 | 0.95-1.84 | 0.085 |
| **HCT-CI** |  |  |  |  |  |  |  |  |  |  |  |  |  |  |  |
| 0 | 1 |  |  |  | 1 |  |  |  | 1 |  |  |  | 1 |  |  |
| 1-2 (without hepatic comorbidity) | 1.43 | 1.10-1.85 | 0.008 |  | 1.29 | 0.99-1.68 | 0.056 |  | 1.27 | 0.95-1.68 | 0.107 |  | 1.11 | 0.82-1.47 | 0.499 |
| 1-2 (with hepatic comorbidity) | 2.90 | 2.05-4.10 | <0.001 |  | 2.57 | 1.79-3.62 | <0.001 |  | 2.20 | 1.46-3.32 | <0.001 |  | 1.86 | 1.20-2.79 | 0.004 |
| ≥ 3 (without hepatic comorbidity) | 1.44 | 1.06-1.97 | 0.020 |  | 1.26 | 0.91-1.72 | 0.146 |  | 1.65 | 1.20-2.25 | 0.002 |  | 1.33 | 0.96-1.82 | 0.083 |
| ≥ 3 (with hepatic comorbidity) | 3.46 | 2.48-4.82 | <0.001 |  | 2.66 | 1.86-3.72 | <0.001 |  | 3.35 | 2.31-4.84 | <0.001 |  | 2.68 | 1.81-3.89 | <0.001 |
| **Disease risk** |  |  |  |  |  |  |  |  |  |  |  |  |  |  |  |
| Standard | 1 |  |  |  | 1 |  |  |  | 1 |  |  |  | 1 |  |  |
| High | 2.19 | 1.78-2.70 | <0.001 |  | 1.76 | 1.41-2.20 | <0.001 |  | 1.95 | 1.57-2.44 | <0.001 |  | 1.40 | 1.11-1.77 | 0.005 |
| Other | 1.42 | 0.84-2.39 | 0.191 |  | 0.99 | 0.53-1.73 | 0.985 |  | 0.38 | 0.14-1.03 | 0.058 |  | 0.54 | 0.16-1.31 | 0.235 |
| **Number of transplants** |  |  |  |  |  |  |  |  |  |  |  |  |  |  |  |
| 1 | 1 |  |  |  | 1 |  |  |  | 1 |  |  |  | 1 |  |  |
| ≥ 2 | 2.10 | 1.70-2.60 | <0.001 |  | 1.59 | 1.25-2.03 | <0.001 |  | 1.30 | 1.00-1.69 | 0.046 |  | 1.10 | 0.82-1.46 | 0.519 |
| **Donor** |  |  |  |  |  |  |  |  |  |  |  |  |  |  |  |
| Matched Related BM & PB | 1 |  |  |  | 1 |  |  |  | 1 |  |  |  | 1 |  |  |
| Mismatched Related BM & PB | 1.74 | 1.12-2.70 | 0.013 |  | 1.13 | 0.69-1.85 | 0.624 |  | 2.98 | 1.54-5.78 | 0.001 |  | 1.69 | 0.83-3.53 | 0.149 |
| Matched Unrelated BM & PB | 1.01 | 0.68-1.51 | 0.946 |  | 1.18 | 0.76-1.85 | 0.451 |  | 1.78 | 0.97-3.27 | 0.064 |  | 1.15 | 0.61-2.29 | 0.672 |
| Mismatched Unrelated BM & PB | 1.07 | 0.70-1.64 | 0.742 |  | 1.20 | 0.75-1.92 | 0.440 |  | 1.84 | 0.97-3.49 | 0.060 |  | 1.17 | 0.60-2.38 | 0.649 |
| CB | 1.64 | 1.16-2.29 | 0.004 |  | 1.32 | 0.90-1.95 | 0.160 |  | 5.80 | 3.42-9.81 | <0.001 |  | 2.78 | 1.61-5.18 | <0.001 |
| Related haplo-PTCY | 1.61 | 1.07-2.46 | 0.024 |  | 1.62 | 1.00-2.63 | 0.052 |  | 3.88 | 2.13-7.08 | <0.001 |  | 2.45 | 1.29-4.91 | 0.008 |
| **Conditioning intensity** |  |  |  |  |  |  |  |  |  |  |  |  |  |  |  |
| RIC | 1 |  |  |  | 1 |  |  |  | 1 |  |  |  | 1 |  |  |
| MAC | 0.87 | 0.72-1.06 | 0.178 |  | 1.32 | 1.02-1.71 | 0.033 |  | 1.41 | 1.13-1.76 | 0.002 |  | 1.93 | 1.45-2.58 | <0.001 |
| **TBI use** | 1.10 | 0.89-1.35 | 0.366 |  | 1.19 | 0.91-1.57 | 0.203 |  | 0.72 | 0.58-0.90 | 0.003 |  | 2.12 | 1.50-3.01 | <0.001 |
| **BU use** | 0.80 | 0.65-0.98 | 0.028 |  | 0.81 | 0.62-1.06 | 0.126 |  | 2.09 | 1.67-2.61 | <0.001 |  | 2.46 | 1.80-3.37 | <0.001 |
| **MEL use** | 1.40 | 1.15-1.71 | <0.001 |  | 1.30 | 1.00-1.68 | 0.050 |  | 2.26 | 1.81-2.83 | <0.001 |  | 2.72 | 1.97-3.79 | <0.001 |
| **GVHD prophylaxis** |  |  |  |  |  |  |  |  |  |  |  |  |  |  |  |
| CSA-based | 1 |  |  |  | 1 |  |  |  | 1 |  |  |  | 1 |  |  |
| TAC-based | 1.02 | 0.78-1.33 | 0.893 |  | 0.80 | 0.59-1.08 | 0.125 |  | 2.96 | 1.93-4.52 | <0.001 |  | 1.95 | 1.26-3.18 | 0.005 |
| Other | 1.41 | 0.75-2.62 | 0.285 |  | 1.16 | 0.58-2.13 | 0.647 |  | 1.86 | 0.70-4.92 | 0.213 |  | 1.64 | 0.54-4.10 | 0.331 |
| **HBV** |  |  |  |  |  |  |  |  |  |  |  |  |  |  |  |
| Negative | 1 |  |  |  | 1 |  |  |  | 1 |  |  |  | 1 |  |  |
| Positive | 1.52 | 0.67-3.46 | 0.317 |  | 1.43 | 0.56-3.02 | 0.397 |  | 1.51 | 0.62-3.71 | 0.367 |  | 1.34 | 0.46-3.03 | 0.536 |
| **HCV** |  |  |  |  |  |  |  |  |  |  |  |  |  |  |  |
| Negative | 1 |  |  |  | 1 |  |  |  | 1 |  |  |  |  |  |  |
| Positive | 2.28 | 1.23-4.22 | 0.009 |  | 2.26 | 1.14-4.04 | 0.011 |  | 0.75 | 0.24-2.37 | 0.628 |  | 0.68 | 0.17-1.83 | 0.511 |

*OR* odds ratio*, CI* confidence interval*, ECOG* Eastern Cooperative Oncology Group*, PS* performance status, *HCT-CI* hematopoietic cell transplantation comorbidity index, *BM* bone marrow, *PB* peripheral blood, *CB* cord blood, *PTCY* posttransplant cyclophosphamide, *RIC* reduced-intensity conditioning, *MAC* myeloablative conditioning, *TBI* total body irradiation, *BU* busulfan, *MEL* melphalan, *CY* cyclophosphamide, *GVHD* graft versus host disease, *TAC* tacrolimus, *CSA* cyclosporine, *HBV* hepatitis B virus, *HCV* hepatitis C virus.
